# Prevalence and genetic etiology of poly-cystic ovarian syndrome (PCOS) in Mauritania

**DOI:** 10.3389/frph.2025.1461405

**Published:** 2025-06-09

**Authors:** Marieme Elwafi, Abdi Ahmed, Omar Akhouayri, Ahmed Zein, Hamma Abdelkader, Roughaya Selman, Ahmed Houmeida

**Affiliations:** ^1^ Biomarker Research Unit for Mauritanian Populations, Faculty of Science and Technology, Nouakchott University, Nouakchott, Mauritania; ^2^ Gynecology Department, Faculty of Medicine, Nouakchott University, Nouakchott, Mauritania; ^3^ Department of Obstetrics and Gynecology, El Manar Clinic, Nouakchott, Mauritania; ^4^ Biology and Health Laboratory, Faculty of Sciences Kenitra, Ibn Tofail University, Kenitra, Morocco; ^5^ Department of Obstetrics and Gynecology, Towfik Clinic, Nouakchott, Mauritania

**Keywords:** genetics, prevalence, etiology, PCOS, Mauritania

## Abstract

**Background:**

Polycystic ovary syndrome (PCOS) is a common endocrine and metabolic disorder characterized by polycystic ovaries, oligoanovulation, hyperandrogenism and infertility. The exact specific causes of this disease have not yet been identified, but there is evidence of significant genetic involvement.

**Objective:**

The present study aimed to evaluate the prevalence of PCOS and explore its gene polymorphisms in the Mauritanian population.

**Material and methods:**

Files of 2,100 women patients attending two gynaecologic clinics of Nouakchott were retrospectively analysed to identify PCOS patients based on the 2003 Rotterdam Criterion. A genetic study used Sanger sequencing to search for six known SNPs in LHCGR (rs2293275), FSHR (rs6166), ESR1 (rs2234693), GnRHR (rs104893836), miR-126 (rs4636297), and miR-499 (rs3746444) among 8 familial PCOS cases and 3 sporadic patients. A more extended search was then carried out exclusively for LHCGR rs2293275 on 56 PCOS patients.

**Results:**

The prevalence of PCOS was 7.8% in this cohort. The occurrence of LHCGR rs2293275 (T>C, G; p. Asn 312 Ser) and ESR1 rs2234693 (T>C, G) polymorphisms in the PCOS screened patients suggests a likely association of these variants with the disease. However, rs104893836 polymorphism was not found in any of the tested PCOS cases.

**Conclusion:**

Although yet to be confirmed in larger size cohort, these data could contribute to improving the exploration, referral, and treatment of PCOS in Mauritania.

## 1 Introduction

Polycystic ovary syndrome (PCOS) is an endocrine disorder characterized by dysfunction of the ovaries, affecting 15%–20% of women at childbearing age, with a variable prevalence depending on the diagnostic criteria (1). According to the Rotterdam criteria in 2003, a woman is diagnosed with PCOS if she presents two of the following three conditions: oligoanovulaion or chronic anovulation (O), clinical and/or biological signs suggesting hyperandrogenism (H), and polycystic ovaries observed through ultrasound examination (P) (2). PCOS syndrome could have long-term consequences, such as infertility, endocrine, metabolic, neurological, psychological disorders but also complications during pregnancy, obesity, type 2 diabetes, cardiovascular and ontological diseases (3). In addition, this syndrome was associated with high levels of depression (4).

Despite these health risks, PCOS etiology remained poorly understood, and several hypotheses on the origin of the disease have been proposed (5, 6). Studies on the genetic predisposition to PCOS suggested an oligogenic condition with a heritability of 70% (7). Overall, 241 genes and 144 SNPs were far associated with PCOS (8). These genes are mainly linked to synthesis of proteins belonging to six categories, according to their pathophysiological function: secretion or action of gonadotropin, biosynthesis or function of steroid hormones, production or insulin signaling, insulin resistance or type 2 diabetes, obesity or dyslipidemia, and chronic inflammatory reactions (6).

Despite its global impact, data regarding PCOS in Mauritania remained scarce and, to our knowledge, there is no published data on this pathology in Mauritania. This first study aimed therefore to assess the prevalence of PCOS and explore its genetic background in the Mauritanian population. We selected the genes studied based on their documented involvement in the pathophysiology of PCOS worldwide, including gonadotropin secretion and actions (GnRHR, LHCGR, and FSHR), transient and sexual development (ESR1), and cell proliferation (miR-126, miR-499).

## 2 Material and methods

### 2.1 Epidemiological study

We collected and analyzed the medical records of 2,100 women who attended different gynaecologist clinics in Nouakchott, the capital city, from the years 2019 to 2022. The patients belong to different ethnic groups and regions of the country. The diagnosis was based on the Rotterdam 2003 criteria, which include presence of oligoanovulaion or chronic anovulation (O), clinical and/or biological signs suggesting hyperandrogenism (H), and polycystic ovaries by ultrasound examination (P). Patients' demographic parameters and clinical characteristics of PCOS were collected whenever available.

### 2.2 Genetic study

This study was carried out in two phases. In the first stage, we selected six SNPs: LHCGR (rs2293275), FSHR (rs6166), ESR1 (rs2234693), GnRHIR (rs104893836), miR-126 (rs4636297), and miR-499 (rs3746444) based on their previously reported associations with polycystic ovary syndrome (PCOS) in the literature and their potential functional significance in reproductive endocrinology. These SNPs were first genotyped in a small group of 11 PCOS patients of which 8 patients had positive family hoistory of the disease, in order to increase the likelihood of identifying relevant genetic variants. Based on the results of this exploratory study, we selected the most promising SNP here (LHCGR (rs2293275) which was then investigated in a larger cohort i.e., 56 patients. As the genetic polymorphisms targeted here may be associated with other hereditary coexisting conditions i.e., uterine fibroids within families, we have, in coordination with the gynecologist, limited our cohort to patients whose medical record stated unambiguously only PCOS syndrome.

After a written informed consent, venous blood samples were collected on an EDTA tube. Genomic DNA was extracted using the Qiagen kit according to the procedure joined to the kit. DNA quality and concentration were assessed using agarose gel electrophoresis and Nanodrop spectrophotometry.

Briefly, PCR reactions (20 µl) contained 1 µl (20–30 ng) of genomic DNA, 1 µl (10 µM) of specific primers, 10 µl of Amplitaq Gold 360 Master Mix (Life Technologies) and 8 µl of distilled water. The amplification program presented a DNA denaturation step at 95°C for 10 min followed by 35 cycles of (denaturation at 94°C for 30 s, annealing at 53°C for 35 s, and extension at 72°C for 40 s) tailed by a 5 min final extension at 72°C. To explore the six (6) polymorphisms, PCR reactions were carried out using primers shown in Table 1. The quality of PCR products was checked by 2% agarose gel electrophoresis. The amplicons were purified on a membrane before fluorometric quantification using PicoGreen reagent (Invitrogen). All validated samples were then sequenced using an ABI 3730XL DNA sequencer by Genoscreen (Genoscreen, Inc., Lille, France). Sequencing data obtained were then matched with reference sequences using Seqscape3.0 software program package (Gene Codes, MI, and USA).

**Table 1:** Primers of the genotyping of six SNP.

Gene	SNP	Allele	Sequences of primers	PCR product
ESR1	rs2234693	T>C, G	F: 5’ GGGTTATGTGGCAATGACGT 3’	164 bp
			R: 5’ GACCAATGCTCATCCCAACTC 3’	
FSHR	rs6166	C>T	F: 5’ -CCC AAA TTT ATA GGA CAG-3’	114 bp
			R: 5'-GAG GGACAA GTA TGT AAG TG-3	
GnRHR	rs104893836	T>C	F: 5′ AGATCCGAGTGACGGTTACTTT-3′	530 bp
			R: 5′-CTGTCCGACTTTGCTGTTGCTT-3′	
LHCGR	rs2293275	T>C, G	F: 5’ -CCTCTTCTCTTTCAGACAGA-3’	111 bp
			R: 5’ -CATGCAAATACTTACAGTGTTTTGGTA-3'	
miR-126	rs4636297	A>G, T	5’-CCCGGAGCCTCATATCAGC-3’	285 bp
			5’-GCTATGCCGCCTAAGTACGTC-3’	
miR-499	rs3746444	A>C, G	5’-GCCCCTTGTCTCTATTAGCTG-3’	416 bp
			5’-ACTTTTGCTCTTTCACTCTCAT-3’	

## 3 Results

### 3.1 Epidemiological study

**Figure 1:**
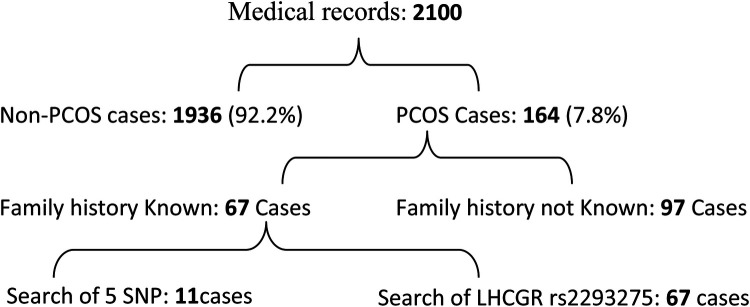
Flowchart of patient selection and genotyping.

**Figure 2:**
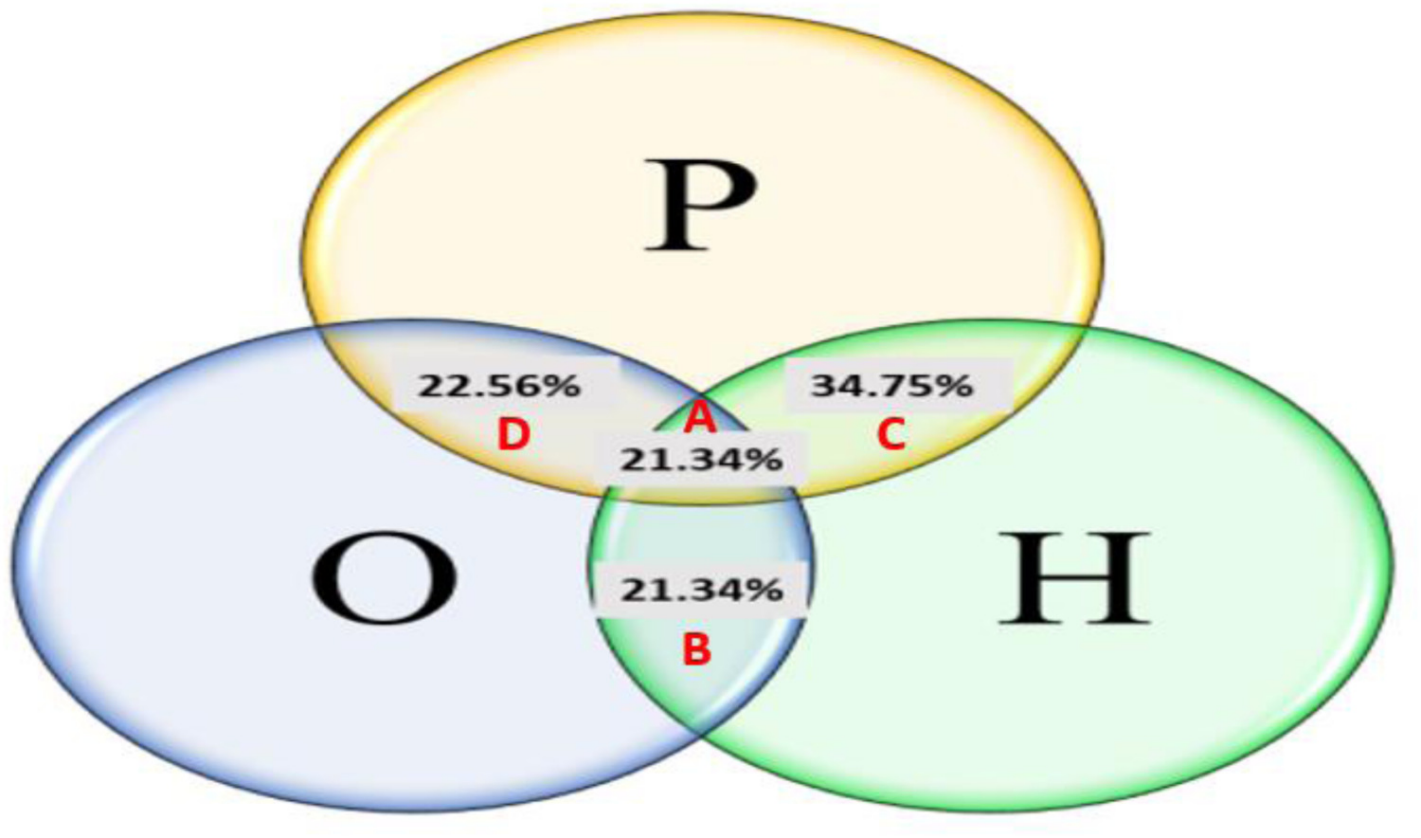
Distribution of PCOS phenotypes among the carrier population.

Out of the 2,100 patient files attending the different selected gynaecologist clinics, 164 women were identified as carrying common PCOS symptoms representing a global prevalence of 7.8% (Figure 1). The cases were distributed into 4 PCOS phenotypes: phenotype-A (H + O + P) (21.34%), phenotype-B (H + O) (21.34%), phenotype-C (H + P) (34.75%) and phenotype-D (O + P) (22.56%). It is noteworthy that phenotype C was the most common, followed by phenotypes B and D (Figure 2).

### 3.2 Genetic study

**Figure 3:**
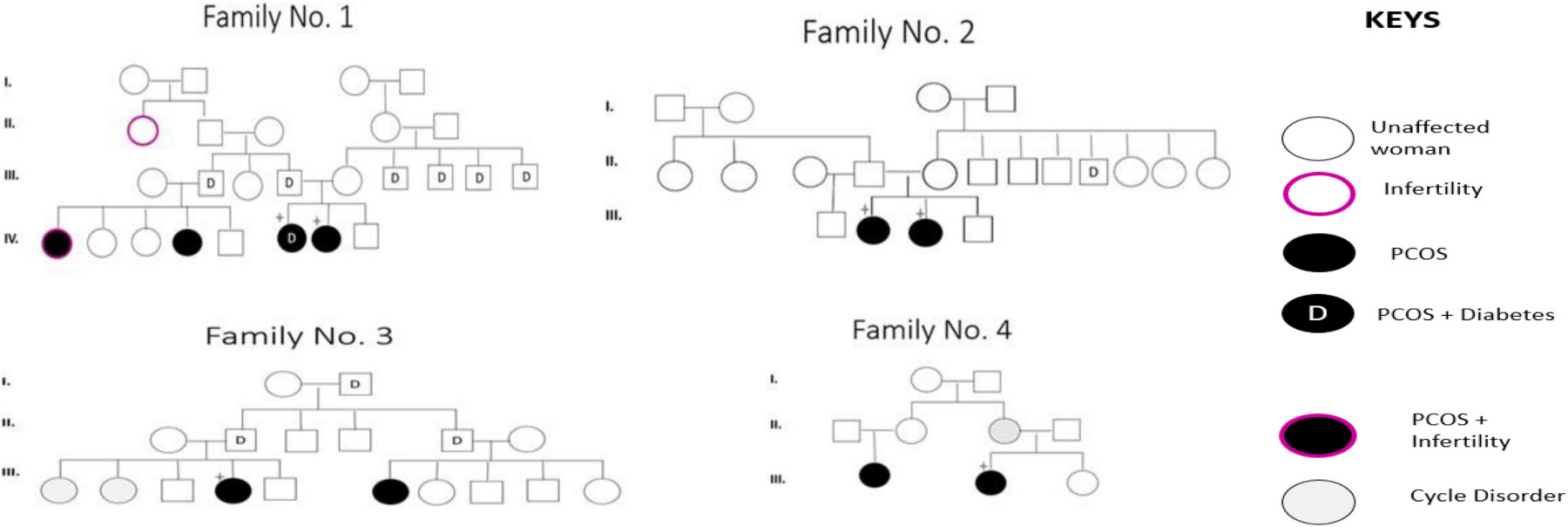
Pedigrees of affected available PCOS families.

Although some patients report a family history of PCOS, this does not necessarily imply the presence of a carrier family member. In the present study, out of the 67 PCOS patients who knew whether the disease was in their families, 14 (20.8%) reported having an affected relative. Among the 11 patients selected for screening of the 6 SNPs, eight had family history of PCOS and 3 were sporadic cases. The pedigrees of four of the six identified families are shown in Figure 3.

In the preliminary screening, Sanger sequencing of DNA from 11 of these patients revealed differences in the occurrence of the six targeted SNPs (Table 2). The highest allele frequency (19 out of 22, 86%) was observed with LHCGR rs2293275 (T>C, G; p.Asn 312 Ser) variant. This variant was found in a homozygous mutant state in 5 familial PCOS and in all 3 sporadic cases.

**Table 2:** Sequencing results of six SNPs in 11 PCOS cases.

Polymorphisms	Family cases								Sporadic cases		
	S1	S2	S3	S4	S5	S6	S7	S8	S9	S10	S11
Rs2293275 (T>C, G)	TC	TC	CT	CC	CC	CC	CC	CC	CC	CC	CC
Rs2234693 (T>C, G)	CC	CC	TC	CC	TC	TT	CC	TC	TC	TC	TC
Rs6166 (C>T)	CT	CT	CT	CT	CT	CT	CC	TT	CT	TT	TT
Rs4636297 (A>G, T)	AG	AG	AG	AG	AA	GG	AA	AA	AG	AG	AG
Rs3746444 (A>C, G)	AA	AA	AA	AA	AA	AA	AA	AA	AG	AA	AA
Rs104893836 (T>C)	TT	TT	TT	TT	TT	TT	TT	TT	TT	TT	TT

The three remaining patients with a family history were heterozygous for this variant. No wild (normal) genotype (TT) was observed. When the analysis of this variant was extended, we also observed a high proportion of patients carrying the mutant allele, both at homozygote genotype (30/56) and heterozygote state (18/56). Globally, looking at the whole cohort (11 + 56 PCOS patients) where this SNP was investigated, we obtained an allele frequency of the mutant allele of 72% (Table 3).

**Table 3:** Genotype and frequency of mutant allele.

SNP	number of PCOS cases	Wild genotype	Homozygote genotype	Heterozygote genotype	frequency of mutant allele
Rs2293275	67	8	38	21	0.72
Rs2234693	11	1	4	6	0.63
Rs6166	11	1	3	7	0.59
Rs4636297	11	3	1	7	0.4
Rs3746444	11	10	0	1	0.04
Rs104893836	11	11	0	0	0

This SNP was followed by ESR1 rs2234693T>C, G with an allele frequency of 14/22. It was in a homozygous state in 4 patients (family cases) and in a heterozygous state in 6 patients (3 families and 3 sporadic cases). The third variant in order of prevalence, was FSHR rs6166 (C.2039 A>G; p.Asn 680 Ser) with an allele frequency of (13/22). It was in a homozygous state in 3 patients (1 family and 2 sporadic cases) and in a heterozygous state in 7 patients (6 family cases and 1 sporadic case).

Two intronic variants were identified: miR-126 rs4636297 (A>G, T) had an allelic frequency of 9/22, with a homozygous mutant state in one familial case, 7 heterozygous genotypes (in 4 familial cases and 3 sporadic cases), as well as a normal genotype (AA) in three familial cases. As for miR-126 rs3746444 A>C, G, it was in a heterozygous state only in a patient with Polycystic Ovary Syndrome (PCOS).

## 4 Discussion

PCOS prevalence variations have been attributed to numerous factors such as sample size, diagnostic criteria, and socioeconomic differences, but also to population location and ethnic background. This study, the first carried out in Mauritania to assess PCOS prevalence and genetic profile, aimed at a representative population of 2,100 women out of a general population of about 4 million inhabitants (9). The percentage we found (7.8%) was within the average PCOS global prevalence of 5%–20% reported by numerous systematic reviews and meta-analysis worldwide (10). Large sample size cross-sectional surveys have indeed reported a prevalence of 3%, 4%, and 10% in Iran, USA, and the UK respectively, while in Australia, Turkey, and Denmark a higher rate (15%–20%) was found (11). Although the number of studies on PCOS in Africa remained limited, the prevalence also varied between 8.2% and 22.5% (12). In our study, we used the Rotterdam criteria diagnostic, which takes into account broader evidence of the disease (oligomenorrhea/amenorrhea, clinical/biochemical hyperandrogenism, and polycystic ovaries) (2). The age of PCOS patients in our cohort ranged from 16 to 40 years, which fits with the reproductive age of women in our population. It was also similar to that reported in the Middle East and North Africa region, which peaked in the 20–24 age group and decreased rapidly from 45 years of age (13).

Gene association studies have so far identified 16 candidate loci involved in PCOS, including genes involved in gonadotropin action (LHCGR and FSHR), metabolism and sexual development (ESR1), insulin signaling and type 2 diabetes (INSR, THADA, HMGA2) or cell proliferation (YAP1 and SUMO1P1) (14).

In the preliminary analysis, we have focused on six of the most prominent SNPs associated with PCOS pathogenesis (15, 16). Among 11 patients and using Sanger DNA sequencing, we observed differences in the occurrence of the six targeted SNPs, with the highest allele frequency (19/22) in LHCGR rs2293275 (T>C, G; p.Asn 312 Ser). When the analysis of this variant was extended to 56 other PCOS patients, the mutant allele was at the homozygous genotype in 30/56 and in the heterozygous state in 18/56, respectively. Globally, looking at the whole cohort (11 + 56 PCOS patients) where this SNP was investigated, we obtained an allele frequency of the mutant allele of 72%. The LHCGR gene encodes luteinizing hormone/choriogonadotropin receptor, whose activation is required during reproduction (16). Although we did not perform a case-control study here due to funding constraints, the proportion of the homozygous genotype and familial cases of this SNP is in agreement with different studies reporting a higher prevalence of this variant in PCOS patients compared to controls, suggesting that this nonsynonymous substitution (p.asn31ser) may reasonably increase the risk of PCOS in women of reproductive age (17). For instance, a case- control analysis in Punjabi populations showed that the mutant homozygous genotype and mutant allele of rs2293275 conferred a 1.7 and 1.3 fold risk respectively towards PCOS progression (18). A similar comparative study concluded that the variant rs2293275 in exon 10 of LHCGR confers a risk for PCOS in both Sardinian (19) and Egyptian populations (20). This variant results in a substitution of the amino acid at position 312 (p.Asn312Ser), near glycosylation sites critical for the stability, trafficking, and expression of the G-protein-coupled receptor superfamily (17). This proximity to glycosylation sites may explain the higher risk of developing PCOS in carriers of this SNP compared to non-carrier.

Our study also revealed that variants rs2234693 (T>C, G) in ESR1 (Estrogen Receptor 1) and rs6166 (C.2039 >G; p.Asn680Ser) in FSHR genes had similar allele frequencies, i.e, 14/22 and 13/22 respectively, among the PCOS patients screened. This result was in agreement with the estrogen action in mediating ovarian folliculogenesis and ovulation (21). However, while rs2234693, rs9340799 and rs8179176 in ESR1 gene and rs4986938 in ESR2 gene were significantly associated with PCOS in Pakistani women (22), no significant associations between the variants of ESR1 rs2234693, ESR1 rs9340799 were observed in a Caucasian population (23). Similarly, the FSHR rs6166 polymorphism did not influence the risk of developing PCOS in a retrospective observational study comparing a PCOS group (*n* = 88) with a control group (*n* = 80) of European women (24).

Unlike the other variants, GNRHR rs104893836T>C (p.Gln106Arg) was absent in all PCOS cases we explored in this preliminary study. This result suggests that this mutation may not be involved in PCOS pathogenesis in our population, although we cannot exclude a possible interaction of this variant with the genetic background and/or environment factors of PCOS in the absence of case-control study (15).

Polycystic ovaries and uterine fibroids are conditions that cause growths in or on the female reproductive organs and share overlapping clinical features such as hormonal dysregulation, estrogen dependence, and metabolic disturbances which can affect fertility. Besides, both conditions may share common genetic or epigenetic pathways and often coexist in women. By limiting the genetic analysis to patients with ambiguously only PCOs syndrome, the variants we found are therefore likely associated with PCOS condition in our population.

However, as inconsistencies in the association of candidate gene polymorphisms with PCOS disease were largely attributed to sample size and ethnic origin (24), our data will still need confirmation by case- control studies including significantly wider cohorts of patients and healthy controls. Indeed, as in the present study where 97 of the 164 PCOS patients do not know if there is a PCOS case in their family, it was reported that about 50% of women are not aware that they have polycystic ovary syndrome (PCOS) (25).

### 4.1 Limitations

This study included a limited number of patients and did not perform a case-control approach to reach a statistically significant *p*-value. Additionally, the total levels of hormones such as testosterone and luteinizing hormones were not recorded in the patient's files, which are known to be higher in PCOS cases.

## 5 Conclusion

This first exploratory study on PCOS in Mauritania revealed the presence of the disease in a proportion consistent with the global trend, underscoring its significance as a public health concern. Our results also supported an association between certain candidate gene SNPs, such as LHCGR SNPs rs2293275 and rs2234693T>C, G in ESR1. Large-scale screening should be conducted to confirm the role of these genetic variants in predisposing Mauritanian women to PCOS. It is important to bear in mind that genetic polymorphisms alone may not fully account for the syndrome's development. As a result, future studies on PCOS ought to investigate other potential grounds for this disease, for instance, environmental risk factors.

## Data Availability

The datasets presented in this study can be found in online repositories. The names of the repository/repositories and accession number(s) can be found in the article/Supplementary Material.
